# Laparoscopic enucleation vs. pancreatectomy for small pancreatic neuroendocrine neoplasms: long-term functional and oncological outcomes

**DOI:** 10.1007/s00464-025-11935-7

**Published:** 2025-08-29

**Authors:** Takayuki Miura, Shuichi Aoki, Shimpei Maeda, Masaharu Ishida, Masamichi Mizuma, Kiyoshi Kume, Keigo Murakami, Atsushi Masamune, Toru Furukawa, Takashi Kamei, Michiaki Unno

**Affiliations:** 1 Department of Surgery, Tohoku University Graduate School of Medicine, 1-1 Seiryo-machi, Aoba-ku, Sendai, 980-8574 Japan; 2 Department of Gastroenterology, Tohoku University Graduate School of Medicine, Sendai, Japan; 3 Department of Investigative Pathology, Tohoku University Graduate School of Medicine, Sendai, Japan

**Keywords:** Laparoscopic enucleation, Laparoscopic regular pancreatectomy, Pancreatic neuroendocrine neoplasms, Endocrine insufficiency

## Abstract

**Background:**

Laparoscopic surgery is being increasingly used for pancreatic neuroendocrine neoplasms (PanNENs). Although laparoscopic regular pancreatectomy (LRP) is frequently performed, laparoscopic enucleation (LE), a parenchyma-sparing technique, may be better in preserving pancreatic endocrine function. However, limited evidence is available regarding the long-term oncological and endocrine outcomes of these two laparoscopic methods. This study aimed to compare the surgical and prognostic outcomes and endocrine function preservation in patients undergoing LE and LRP for well-differentiated, non-invasive PanNEN.

**Methods:**

This retrospective cohort study included 67 consecutive patients who underwent laparoscopic surgery for small (< 2 cm) well-differentiated PanNEN at Tohoku University Hospital between January 2001 and December 2021. LE was performed for small tumors (< 2 cm) located away (> 3 mm) from the main pancreatic duct. Clinical characteristics, surgical details, tumor characteristics, postoperative complications, recurrence-free survival (RFS), overall survival (OS), and long-term endocrine function were retrospectively analyzed. Kaplan–Meier analysis, Cox regression, and the Mann–Whitney U test were used for statistical comparisons.

**Results:**

The median follow-up was 78.1 months for LRP and 135.4 months for LE. No significant differences were observed between the two groups in terms of the operative time, blood loss, or postoperative complications. Five-year RFS was excellent and comparable in both groups (LE, 100%, LRP 96.0%; *P* = 0.313). Notably, LE was associated with a significantly reduced incidence of postoperative new-onset diabetes mellitus (NODM) compared to LRP (5-year cumulative incidence: 9.1% vs. 43.2%; *P* = 0.0181). Multivariate analysis identified LRP (hazard ratio [HR] = 7.71, 95% confidence interval [CI]:1.03–57.8; *P* = 0.0469), older age (> 60 years) (HR = 4.49, 95% CI 1.62–12.4; *P* = 0.0039), and non-functional tumor (HR = 2.78, 95% CI 1.08–7.19; *P* = 0.0342) as independent predictors of NODM.

**Conclusion:**

Given appropriate patient selection, LE of small well-differentiated PanNENs provides comparable oncological outcomes, perioperative safety, and superior long-term endocrine function preservation compared to LRP.

**Supplementary Information:**

The online version contains supplementary material available at 10.1007/s00464-025-11935-7.

Pancreatic neuroendocrine neoplasms (PanNENs) are being increasingly diagnosed due to advancements in imaging modalities over the past few decades [1]. These tumors are often discovered incidentally and exhibit a variety of biological behaviors ranging from benign to highly aggressive. Appropriate surgical management is essential for optimizing oncological outcomes, preserving pancreatic function, and minimizing surgical morbidity [2]. Enucleation offers the advantage of preserving endocrine and exocrine pancreatic function by avoiding extensive parenchymal resection. Previous studies have shown that enucleation is associated with a shorter operative time, less blood loss, and similar overall morbidity and long-term outcomes compared to regular pancreatectomy, including pancreaticoduodenectomy (PD) and distal pancreatectomy (DP) [3–6].

Minimally invasive surgery (MIS) is being increasingly used for the resection of pancreatic tumors, including PanNENs [7]. Compared to open surgery, laparoscopic approaches have proven benefits such as reduced intraoperative blood loss, shorter hospital stay, and faster postoperative recovery [8, 9]. Among laparoscopic techniques, enucleation has emerged as a viable alternative to conventional resection, particularly for small, well-differentiated, non-invasive PanNENs.

However, direct comparative studies evaluating oncological outcomes and long-term endocrine function after laparoscopic enucleation (LE) versus laparoscopic regular pancreatectomy (LRP) for PanNEN are lacking.

This study compared the perioperative, short-term, and long-term outcomes of LE to those of LRP for PanNEN. By analyzing postoperative outcomes and endocrine function preservation after surgery, we sought to provide evidence-based recommendations for selecting the optimal surgical approach for managing small, well-differentiated, non-invasive PanNENs.

## Materials and methods

### Patients

This retrospective cohort study was conducted at Tohoku University Hospital and included consecutive patients who underwent laparoscopic resection for well-differentiated PanNEN between January 2001 and December 2021. Patients who underwent either LRP, including laparoscopic distal pancreatectomy (LDP) and laparoscopic pancreaticoduodenectomy (LPD), and LE were enrolled. All procedures were performed by surgeons with extensive experience in pancreatic surgery. The tumor size and Ki-67 expression of the excised specimen were assessed to make the final pathological diagnosis. Patients with the following conditions were excluded from the analysis: the presence of other malignant neoplasms, main vascular invasion by the tumor, multiple tumors associated with or without hereditary syndromes, preoperative distant metastases, or history of pancreatic resection. Central pancreatectomy was excluded from the study because it is rarely performed at our institution and when undertaken, is typically carried out via open surgery. In addition, although central pancreatectomy usually preserves more pancreatic tissue than DP or PD, it has a distinct complication profile. Therefore, including it in the analysis and directly comparing it with LE or LRP would not be appropriate. Eligible patients were classified into two groups based on the type of initial surgery. This study was approved by the institutional review board (IRB) of Tohoku University (approval number: 2024–1-200), and conducted according to the ethical principles outlined in the 1964 Declaration of Helsinki and its later amendments. This study adheres to the Strengthening the Reporting of Observational Studies in Epidemiology (STROBE) guidelines. The requirement for informed consent was waived by the IRB because of the retrospective study design. Clinical data, including patient demographics, tumor characteristics, surgical details, and postoperative outcomes, were retrospectively collected from the medical records. Finally, 67 patients who underwent LRP (*n* = 55) or LE (*n* = 12) were included in this study.

### Surgical procedures

Small and peripheral PanNENs were treated with either LE or LRP. The decision to perform LE or LRP was made preoperatively based on detailed imaging evaluation, including high-resolution computed tomography (CT) and magnetic resonance imaging (MRI). LE was selected for tumors < 2 cm in diameter and located more than 3 mm from the main pancreatic duct. These parameters were confirmed using intraoperative ultrasound, which was routinely employed to verify the tumor location and relationship with the main pancreatic duct. LE involved excision of the tumor and its surrounding capsule from the pancreatic parenchyma using a combination of monopolar hook electrode and Harmonic scalpel (Ethicon Endo-Surgery, Cincinnati, OH, USA), and lymph node sampling when necessary. For a tumor located in the pancreatic head, the duodenum was Kocherized and mobilized to expose the lesion fully. For buried tumors within the parenchyma, a cross-stitch was placed through the tumor to facilitate retraction and enhance exposure from the appropriate angle. Hemostasis was achieved using hemostatic agents or soft coagulation. At the end of the procedure, a drain was routinely placed near the enucleation area of the pancreas. LRP included LDP with or without splenectomy for body and tail lesions and LPD for head lesions, following the established laparoscopic surgical techniques. Each patient underwent the surgery recommended by a multidisciplinary team adhering to the JNETS guidelines [10].

### Follow-up

All patients underwent regular follow-up after the operation, and all follow-up data were collected and analyzed using the same standard criteria and procedures. The first follow-up was conducted within 1 month of the operation, and the subsequent follow-up cycle lasted for 6–12 months. Postoperative surveillance including imaging and serological examinations. The endocrine function status was assessed through outpatient visits and telephone interviews. The patients themselves monitored their HbA1c and fasting blood glucose (FBG). The final follow-up was conducted in February 2025.

### Outcomes and definitions

The primary outcome measures were recurrence-free survival (RFS) following surgery and long-term preservation of endocrine function. The following baseline clinical characteristics of all patients were collected and compared: age, sex, body mass index (BMI), preoperative diabetes mellitus, American Society of Anesthesiologists physical status (ASA-PS), tumor size, tumor location, morphology, World Health Organization (WHO) Classification of Tumors [11], European Neuroendocrine Tumor Society (ENETS) TMN classification [12], hormonal function based on associated symptoms or elevated blood levels of hormones, Ki-67 index, lymph node metastasis, lymphatic-vascular invasion, operative time, estimated blood loss, postoperative hospital stay, and postoperative complications including postoperative pancreatic fistula (POPF), postoperative hemorrhage, and new-onset diabetes mellites (NODM), which were assessed based on the International Study Group of Pancreatic Surgery (ISGPS) [13] or Clavien-Dindo grading standards [14]. Open conversion was defined as when resection was initially attempted laparoscopically but required an open incision to complete the surgery. Recurrence was defined as a new pathologically or radiologically diagnosed hallmark of PanNEN, including local reappearance in the pancreas, lymph node involvement, or distant metastases. RFS was defined as the time from the initial surgery to tumor recurrence or death. Overall survival (OS) was defined as the interval between the surgery and death or the date of last follow-up before February 2025. NODM was defined as postoperative HbA1c ≥ 6.5% or FBG level ≥ 126 mg/dl [15]. Twelve patients diagnosed with diabetes mellitus before the surgery were excluded from this study to determine the incidence and risk factors for NODM.

### Statistical analysis

Continuous variables are expressed as medians (interquartile ranges), while categorical variables are reported as counts and percentages. Differences in continuous variables between the LE and LRP groups were analyzed using the Mann–Whitney U test, whereas categorical variables were compared using Pearson's chi-square test. Kaplan–Meier survival analysis was used to evaluate RFS, OS, and cumulative incidence of diabetes, and differences were assessed using the log-rank test. Risk factors for incident NODM were identified using Cox regression analysis, and are presented as hazard ratios (HRs) and 95% confidence intervals (CI). A *P*-value < 0.05 was considered statistically significant. All statistical analyses were performed using JMP Pro 17.0.0 (SAS Institute Inc., Cary, NC, USA) and GraphPad Prism 10.4.1 (San Diego, CA, USA).

## Results

### Baseline characteristics

Table 1 summarizes the baseline demographic characteristics of the patients in both groups. A total of 67 consecutive patients who underwent laparoscopic surgery for PanNEN were included in this study. Among them, 55 patients underwent LRP and 12 underwent LE. The proportion of morphologically protruding type of PanNEN was significantly higher in patients undergoing LE than in patients undergoing LRP (58.3% vs. 3.6%, *P* < 0.001). In contrast, age, sex, BMI, preoperative diabetes mellitus, ASA-PS score, tumor size, tumor location, clinical stage, and hormonal function were comparable between the LRP and LE groups (Table 1). On pathological examination, all patients had low-grade PanNEN (G1: 83.6, 58.3%; G2: 16.4, 41.7%, respectively). Regarding hormonal function, there were 18 insulinomas and one gastrinoma in the LRP group and seven insulinomas in the LE group; and the rest were non-functional tumors.

**Table 1:** Baseline and clinicopathological characteristics of patients

Variables	LRP group (*n* = 55)	LE group (*n* = 12)	*P* value
Age (years)^†^	61.5 ± 14.5	57.4 ± 20.5	0.6352
Sex (male/female)	25/30	5/7	0.8110
Body mass index^†^	23.3 ± 4.0	25.0 ± 3.1	0.1455
ASA–PS			0.2160
1	8 (14.6)	4 (33.3)	
2	34 (50.1)	7 (58.4)	
3	13 (23.6)	1 (8.3)	
Preoperative diabetes mellitus	12 (18.2)	0 (0)	0.1093
Tumor size (mm)	12 (7–17)	11.5 (8.6–14.5)	0.8435
Tumor location			0.1780
Pancreatic head	9 (16.4)	4 (33.3)	
Pancreatic body or tail	46 (83.6)	8 (66.7)	
Morphology			** < 0.001**
Protruding type	2 (3.6)	7 (58.3)	
Buried type	53 (96.4)	5 (41.7)	
WHO classification			0.0667
G1	46 (83.6)	7 (58.3)	
G2	9 (16.4)	5 (41.7)	
TMN Clinical stage			0.758
I	42 (76.4)	10 (83.3)	
II	11 (20.0)	2 (16.7)	
III	2 (3.6)	0	
Hormonal function			0.1255
No	36 (65.4)	5 (41.7)	
Yes	19 (34.6)	7 (58.3)	
Ki–67 (%)	1.6 (1–2.5)	2.1 (0.53–7.3)	0.5542
Lymph node metastasis	2 (3.64)	0 (0)	0.5024
lymphatic–vascular invasion	14 (25.5)	1 (8.3)	0.1974

Data are expressed as number (percentage), median (interquartile range)

*ASA-PS* American Society of Anesthesiologists Physical Status, *BMI* body mass index, *WHO* World Health Organization, *ENETS* European Neuroendocrine Tumor Society, *POPF* postoperative pancreatic fistula, *LRP* laparoscopic regular pancreatectomy, *LE* laparoscopic enucleation

^†^Data expressed as the mean ± standard deviation

*P*-value < 0.05 marked in bold font shows statistical significance

### Perioperative outcomes

Table 2 compares the perioperative outcomes between the LRP and LE groups. None of the patients had preoperative evidence of distant metastasis, which was confirmed surgically. Of the 67 laparoscopic procedures, 45 were LDP (without splenectomy, *n* = 18), nine were LPD, four were enucleations in the pancreatic head, and eight were enucleations in the pancreatic body or tail. No postoperative mortality was observed. The median operative time and estimated blood loss did not differ significantly between the two groups. Regarding postoperative complications, the LE group tended to have a lower incidence of POPF grade ≥ B than the LRP group (8.3% vs. 32.3%; *P* = 0.06). Postoperative hemorrhage, re-admission rate, and length of postoperative hospital stay were similar in both groups.

**Table 2:** Surgical outcomes of patients with PanNEN

Variables	LRP group (*n* = 55)	LE group (*n* = 12)	*P* value
Operative procedures			** < 0.001**
Pancreaticoduodenectomy	9 (16.4)	0 (0)	
Distal pancreatectomy	46 (83.6)	0 (0)	
Enucleation of pancreatic head	0 (0)	4 (33.3)	
Enucleation of pancreatic body and tail	0 (0)	8 (66.7)	
Operative time (min)	421 (317–601)	356.5 (222–424)	0.0517
Estimated blood loss (ml)	102 (21–337)	28 (10–430)	0.1179
Conversion to open surgery	5 (9.1)	2 (16.7)	0.4370
Surgical margin status			0.2295
R0	54 (98.2)	11 (91.7)	
R1	1 (1.8)	1 (8.3)	
POPF ≥ GradeB	18 (32.3)	1 (8.3)	0.0894
Postoperative hemorrhage	1 (1.8)	0 (0)	0.6379
Complication ≥ CD3a	12 (21.8)	1 (8.3)	0.2845
Re-admission within 90 days	1 (1.8)	1 (8.3)	0.2295
Postoperative hospital stay (days)	18 (12–31)	16.5 (10–30.8)	0.5180
New-onset diabetes mellitus	21/45 (46.7)	1/12 (8.3)	**0.0154**
Follow–up period (month)	78.1 (52.2–108.7)	135.4 (53.3–188.8)	0.1110

Data are expressed as number (percentage), median (interquartile range)

*PanNEN* pancreatic neuroendocrine neoplasm, *LRP* laparoscopic regular pancreatectomy, *LE* laparoscopic enucleation, *POPF* postoperative pancreatic fistula

*P*-value < 0.05 marked in bold font shows statistical significance

### Oncological outcomes

**Fig. 1:**
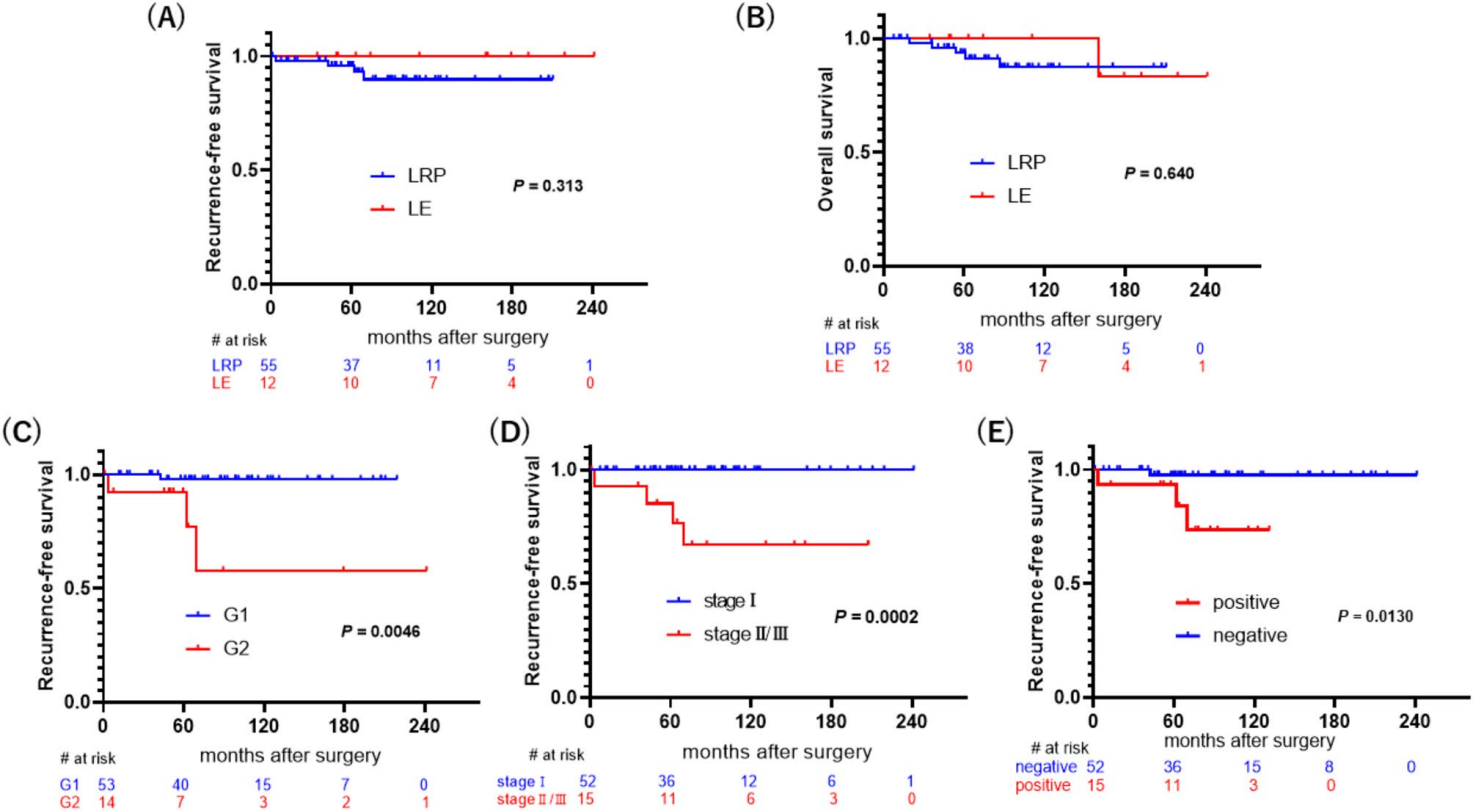
**A** Recurrence-free survival (RFS) in the laparoscopic enucleation (LE) and laparoscopic regular pancreatectomy (LRP) groups (*P* = 0.313). **B** Overall survival (OS) in the LE and LRP groups (*P* = 0.640). **C** Correlation between RFS and the WHO grade (*P* = 0.0046). **D** Correlation between RFS and the TMN clinical stage (*P* = 0.0002). **E** Correlation between RFS and lymphatic-vascular invasion

The median follow-up period was 78.1 months in the LRP group and 135.4 months in the LE group. Forty-seven (70.1%) patients were followed up for at least 5 years. During the follow-up period, four (5.97%) patients had recurrence or metastasis (Supplementary Table 1), and eight patients died (11.9%) due to non-tumor causes, with overall 5-year RFS and 5-year OS rates of 96.8% and 93.7%, respectively. No significant differences in RFS or OS were observed between the LRP and LE groups (Fig. 1A and B). In contrast, a higher WHO grade, TMN clinical stage, and lymphatic-vascular invasion showed a significant correlation with a shorter RFS (*P* < 0.01 for all) after resection (Fig. 1C–E).

### Endocrine function

**Fig. 2:**
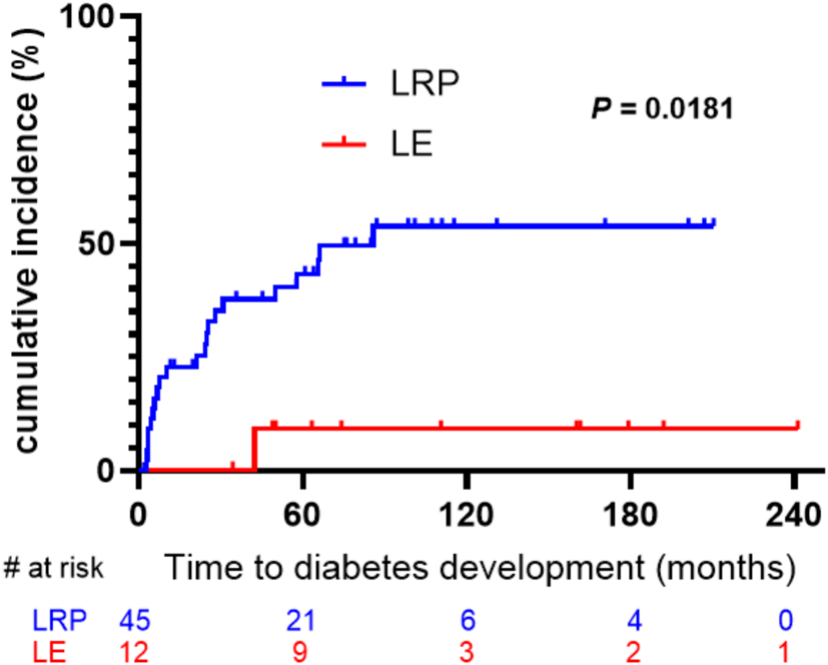
Kaplan–Meier plots of incidence of new-onset diabetes mellitus after surgery. Statistical significance was assessed using the log-rank test, and 95% confidence intervals (95% CI: lower limit, upper limit) were calculated using Cox regression analysis

During follow-up, the incidence of NODM was 46.7% (21/ 45) in the LRP group and 8.3% (1/12) in the LE group (*P* = 0.0154) (Table 2). The cumulative incidence rates of NODM in the LRP and LE groups were 43.2% and 9.1% at 5 years and 53.7% and 9.1% at 10 years, respectively, based on the Kaplan–Meier method and log-rank test (*P* = 0.0181) (Fig. 2). The risk factors for NODM and results of univariate and multivariate analyses for each clinicopathological variable are shown in Table 3. In univariate analysis, the risk of NODM was about eight times higher in patients who received LRP than in those who received LE (95% CI 1.02–56.7, *P* = 0.0474). Additionally, old age (> 60 years) and non-functional PanNEN were significant predictive factors for NODM (*P* < 0.05 for all). In contrast, sex, BMI (> 25), tumor location, WHO classification, and TMN clinical stage did not significantly affect the development of NODM. In multivariate analysis, LRP (HR = 7.71, 95% CI 1.03–57.8, *P* = 0.0469), old age (> 60 years) (HR = 4.49, 95% CI 1.62–12.4, *P* = 0.0039), and non-functional tumor (HR = 2.78, 95% CI 1.08–7.19, *P* = 0.0342) were found to be the independent predictive factors of NODM.

**Table 3:** Risk factors for new-onset diabetes mellites on univariate and multivariate analyses

Variables	Univariate analysis			Multivariate analysis		
	HR	95%CI	*P* value	HR	95%CI	*P* value
Sex			0.3587			
Male	Reference					
Female	0.67	0.29–1.56				
Age (years)			**0.0116**			**0.0039**
≤ 60	Reference			Reference		
> 60	3.67	1.34–10.1		4.49	1.62–12.4	
BMI (kg/m^2^)			0.4116			
≤ 25	Reference					
> 25	1.43	0.61–3.35				
Operative methods			**0.0474**			**0.0469**
LE	Reference			Reference		
LRP	7.62	1.02–56.7		7.71	1.03–57.8	
Function			**0.0322**			**0.0342**
Yes	Reference			Reference		
No	2.81	1.09–7.22		2.78	1.08–7.19	
Tumor location			0.9114			
Pancreatic head	Reference					
Pancreatic body or tail	1.06	0.34–2.87				
WHO classification			0.5641			
G1	Reference					
G2	0.70	0.21–2.36				
TMN clinical stage			0.0917			
I	Reference					
II/III	0.18	0.02–1.32				

*HR* hazard ratio, *CI* confidence interval, *WHO* World Health Organization, *BMI* body mass index

*P*-value < 0.05 marked in bold font shows statistical significance

## Discussion

Laparoscopic pancreatectomy is generally considered more challenging than open pancreatectomy due to the specialized technical requirements and high risk of perioperative complications [16, 17]. This study compared the oncological and endocrine outcomes of LE and LRP for PanNEN over an adequate follow-up duration. Our findings demonstrated that LE is associated with a significantly lower incidence of NODM, while achieving an oncological prognosis comparable to that of LRP. By addressing the long-term metabolic complications, we have highlighted an important consideration in surgical decision-making beyond tumor control alone for low-grade PanNENs. Furthermore, no significant intergroup differences in perioperative complications were observed. The novelty of this study lies in its direct comparison of two laparoscopic approaches in addition to a meticulous evaluation of both oncological and endocrine outcomes, an aspect often overlooked in previous studies. Moreover, few studies have followed up the patients for at least 5 years to evaluate the endocrine outcomes after pancreatectomy. Furthermore, the relatively homogeneous patient cohort and standardized surgical approaches at a single, high-volume center enhanced the reliability of the findings.

Pancreatic endocrine function is an important determinant of the patient's quality of life after surgery. Regular pancreatectomies, such as PD and DP that often involves resection of approximately 50% of the pancreatic volume, are associated with a high incidence of NODM [18, 19]. In contrast, preservation of β-cells in the pancreas during enucleation likely reduces the development of endocrine insufficiency. This observation corresponds with that of Jilesen et al. [20], who documented superior long-term endocrine function after parenchyma-sparing procedures for PanNEN. Additionally, avoiding pancreatic ductal injury and subsequent inflammatory changes in the remnant pancreas may contribute to the superior preservation of endocrine function after enucleation. In this study, the cumulative incidence of NODM was significantly lower with enucleation using a laparoscopic approach, without an increase in the risk of morbidity and recurrence. To our knowledge, no previous studies have demonstrated that LE could reduce the risk of developing NODM compared to LRP. LE significantly correlated with a lower incidence of NODM than LDP and LPD (Supplementary Fig. S1). In multivariate analysis, older age and non-functional PanNEN showed a significant association with the development of NODM. This result reflects the diminished baseline β-cell reserve in older patients. Conversely, it is speculated that the β-cell capability of the normal pancreatic parenchyma may be well retained in patients with insulinoma, which constituted the majority of functional tumors in this study. These results may help to guide clinical decision-making, particularly in patients at high risk of postoperative metabolic disorders.

In this study, the oncological outcomes, specifically RFS and OS, were comparable between the two laparoscopic surgical approaches. This result is consistent with the findings of Weilin et al. [21], who demonstrated that for small well-differentiated PanNENs, parenchyma-sparing procedures could achieve oncological outcomes equivalent to that of standard resections via open surgery. The similar survival outcomes observed in our study likely reflect appropriate patient selection, with laparoscopic enucleation reserved for cases in which complete tumor removal could be achieved while maintaining a safe margin from the main pancreatic duct. Previously, several prognostic indicators for PanNENs, such as the WHO grading, clinical stage, lymph node metastasis, immune-inflammatory markers, and microvascular invasion, have been reported [22–26]. In the present study, Kaplan–Meier survival analysis identified the WHO grade, TNM clinical stage, and lymphatic-vascular invasion as significant factors associated with a shorter RFS. Moreover, our data corroborate the notion that patients with smaller tumors, whether functional or non-functional, tend to have a good prognosis, thus explaining the lack of a significant difference in RFS between the two groups (Supplementary Fig. S2).

The comparable perioperative complication rates between LE and LRP in this study are consistent with those in the previous literature [3, 20], reinforcing that LE does not compromise the short-term safety despite its parenchyma-sparing nature. Although some studies have reported that enucleation has a potentially higher rate of POPF formation [27, 28], a meta-analysis revealed that pancreatic enucleation can be performed with no increased risk of POPF at specialized centers [29]. This finding aligns with that of Hackert et al. [30], who reported that enucleation can be executed with acceptable morbidity rates when performed at high-volume centers with appropriate patient selection. Additionally, in the current study, we strictly selected the cases in which the tumor was located far from the pancreatic main duct (> 3 mm), which may have contributed to the low incidence of clinically relevant POPF (8.3% in the LE group). In the present study, the median operative time was not significantly shorter for LE compared to LRP, in contrast to the findings of previous studies [4, 28]. This lack of difference in the operative time between LE and LRP may be because one-third of the enucleations in this study were performed for tumors located in the pancreatic head, which is known to be a more challenging procedure [31–33], as opposed to most previous studies where enucleations were performed for lesions in the pancreatic body or tail. Additionally, the relatively small proportion of patients who underwent LPD contributed to the equivalence of the operative time between the two groups.

Robotic-assisted surgery, which may further refine enucleation techniques [34], is expected to expand in the future; however, it is not currently covered by Japanese health insurance. Tian et al. [35] conducted robotic enucleation for small PanNENs, which did not increase the rates of POPF or major complications and reduced the estimated blood loss compared to open surgery. Despite this, laparoscopic approaches are likely to persist in many centers for these tumors because of the initial investment and ongoing maintenance costs of robotic systems, which are significant factors worth considering [36]. Our findings suggest that LE can be performed safely with equivalent oncological outcomes and superior endocrine function preservation compared to LRP. Future prospective studies are warranted to validate these findings and establish standardized guidelines for the optimal surgical approach for small PanNENs.

However, this study has several limitations. First, the relatively small sample size, particularly in the LE group, may have limited the statistical power of our findings, necessitating further validation in more extensive multicenter studies. Second, the retrospective nature of this study may have introduced potential selection bias. Although we found no significant differences in the baseline characteristics between the two groups, unmeasured confounders could still influence the outcomes. Third, our study was conducted in a high-volume single center where experienced surgeons performed the procedures; therefore, the results may have limited generalizability.

In summary, the results of this study demonstrate that LE for PanNENs has perioperative and oncological outcomes comparable to those of LRP. Additionally, LE was associated with a lower incidence of NODM, suggesting a favorable impact on long-term endocrine function preservation.

Further prospective multicenter studies are warranted to confirm these findings and refine the patient selection criteria for LE in clinical practice.

## Supplementary Information

Below is the link to the electronic supplementary material.

Supplementary file1 (TIF 85 KB)—Fig. S1 Cumulative incidence of new-onset diabetes mellitus (NODM) stratified by the operative procedure. Fig. S2 Recurrence-free survival associated with hormonal function (*P* = 0.601).

Supplementary file2 (DOCX 32 KB)

## Data Availability

The datasets used and analyzed during this study are available from the corresponding author upon reasonable request.
