# Supplementary figures and images for: Laparoscopic enucleation vs. pancreatectomy for small pancreatic neuroendocrine neoplasms: long-term functional and oncological outcomes
Source: Surg Endosc. 2025 Aug 29;39(11):7407–16. doi: 10.1007/s00464-025-11935-7 (PMC12618291; doi:10.1007/s00464-025-11935-7)

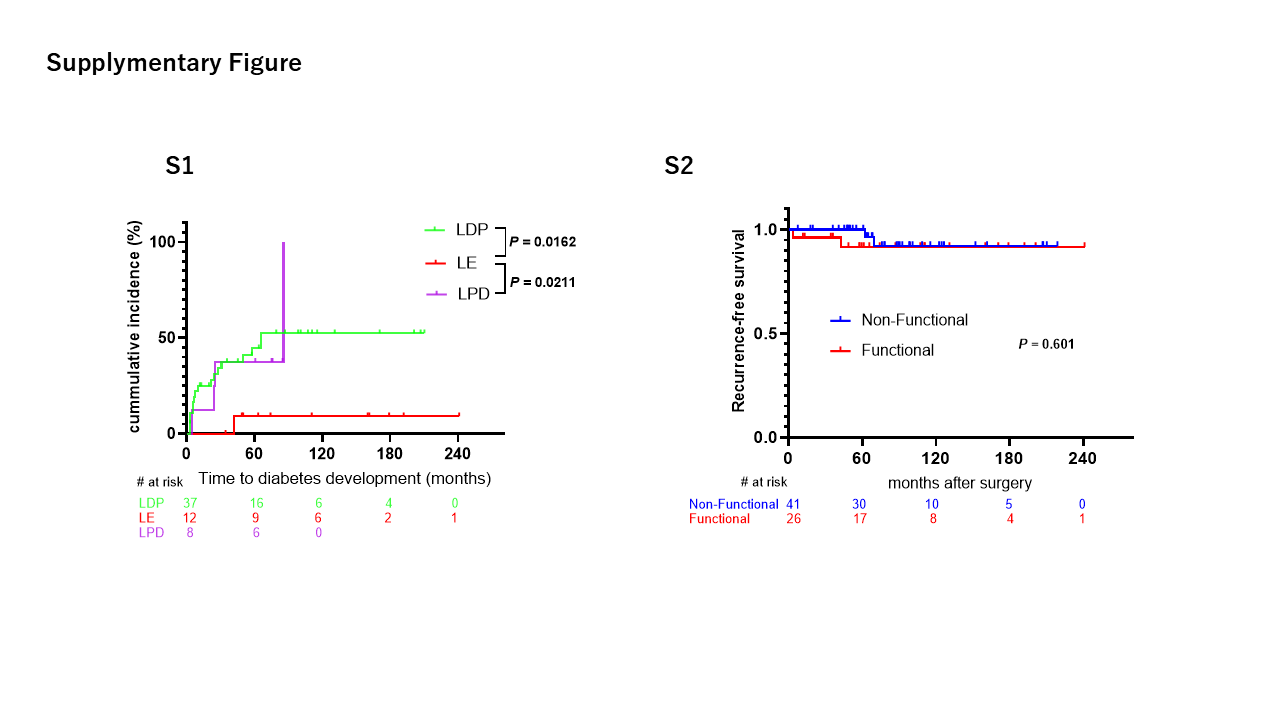

Supplement: Supplementary file 1 — Supplementary file1 (TIF 85 KB)—Fig. S1 Cumulative incidence of new-onset diabetes mellitus (NODM) stratified by the operative procedure. Fig. S2 Recurrence-free survival associated with hormonal function (P = 0.601). [file 464_2025_11935_MOESM1_ESM.tif]
