# Supplementary material for: Laparoscopic enucleation vs. pancreatectomy for small pancreatic neuroendocrine neoplasms: long-term functional and oncological outcomes
Source: Surg Endosc. 2025 Aug 29;39(11):7407–16. doi: 10.1007/s00464-025-11935-7 (PMC12618291; doi:10.1007/s00464-025-11935-7)
Supplement: Supplementary file 2 — Supplementary file2 (DOCX 32 KB) [file 464_2025_11935_MOESM2_ESM.docx]

| **Supplementary table1 Clinicopathological parameters of patients with disease progression** | | | | | | |  |  |  |  |
| --- | --- | --- | --- | --- | --- | --- | --- | --- | --- | --- |
| Age (Years) | Sex | WHO grade | Tumor Location | Tumor size (mm) | Lymph Node | lymphatic-vascular invasion | operation | DFS (months) | site of recurrence | Outcome |
| 62 | female | G2 | pancreatic tail | 20 | Negative | Positive | LDP | 3.4 | Liver | survival |
| 53 | male | G2 | pancreatic tail | 25 | Negative | Positive | LDP | 62.1 | Liver | survival |
| 75 | male | G2 | pancreatic head | 21 | Negative | Positive | LDP | 69.6 | Liver | Death |
| 39 | male | G1 | pancreatic head | 21 | Positive | Negative | LPD | 42.5 | distant lymph nodes | survival |
| LDP, laparoscopic distal pancreatectomy; LPD, laparoscopic pancreaticoduodenectomy | | | | | | |  |  |  |  |
